# Penile self-mutilation preceded by bizarre delusions: two case reports

**DOI:** 10.1186/1752-1947-8-246

**Published:** 2014-07-07

**Authors:** Youssef Kharbach, Driss Amiroune, Mustapha Ahsaini, Amine Bout, Omar Riyach, Roos E Stuurman-Wieringa, Soufiane Mellas, Mohammed Fadl Tazi, Abdelhak Khallouk, Mohammed Jamal El Fassi, Ismail Rammouz, Moulay Hassan Farih

**Affiliations:** 1 Department of Urology, Hassan II University Hospital, Fez, Morocco; 2 Department of Psychiatry, Hassan II University Hospital, Fez, Morocco; 3 Department of Urology, Reinier de Graaf Gasthuis, P.O., GA, Delft, The Netherlands; 4 Department of Anatomy, Faculty of Medicine, Mohammed Ben Abdellah University, Fez, Morocco

**Keywords:** Behavior disorder, Genital self-mutilation, Schizophrenia

## Abstract

**Introduction:**

Genital self-mutilation is listed as a symptom of borderline personality disorder. The type of injury varies from simple skin laceration to total amputation of the penis and testicles. These injuries are urological and surgical emergencies.

**Case presentation:**

We report two cases of penile self-mutilation precipitated by erotic and religious bizarre delusions.

Our first patient is a 24-year-old Moroccan man who visited our emergency room with a metallic ring at the root of his penis which had caused marked edema of his entire penis.

Our second patient is a 26-year-old Moroccan man evaluated in our emergency unit. A clinical examination revealed a wound at the dorsal side of his penis with complete transection of the dorsal vein and imperfect hemostasis.

The two patients were treated in our emergency unit after which a favorable clinical course was observed.

**Conclusion:**

Cases of genital self-mutilation are urological and psychiatric emergencies, therefore it is important that surgical and psychiatric teams collaborate closely while managing cases of genital self-mutilation.

## Introduction

Self-mutilation, self-injuring or self-harming behavior has been defined as a deliberate destruction or alteration of body tissue in the absence of conscious suicidal intention [1].

The most common form of self-mutilation is skin-cutting. Acts of male genital self-mutilation are extremely rare and can cause serious damage to sexual and urinary functions. Since 1846, when the first scientific report was published [2], few cases have been described in the English literature as isolated cases or small series of patients. These mutilations are often observed in young male psychotic patients.

We present two case reports of male genital self-mutilation in patients with schizophrenia.

## Case presentation

### Patient 1

**Figure 1:**
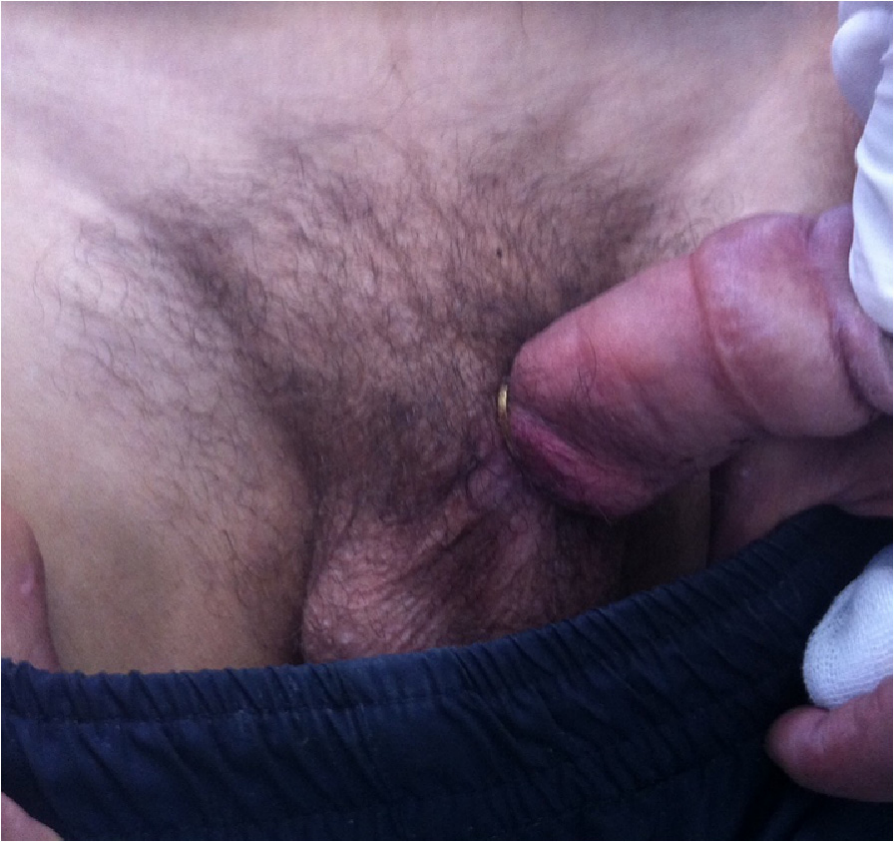
Strangulated penis by a metallic ring at its root.

A 24-year-old Moroccan man with paranoid schizophrenia had stopped his psychiatric medical treatment 2 months before he consulted our emergency unit with an acute urinary retention. He had placed a metallic ring on the root of his penis 7 hours before his admission. On clinical examination he was calm; he was seen with marked edema of his entire penis extending from its root to the glans penis with discrete cyanosis on the glans penis. Multiple rings were also found on his left fingers (Figure 1). He explained that his partner had refused to have sex with him, so he tried to become more “seductive”. These disorganized thoughts caused troubled behavior.

We removed the ring using a ring-cutter, and he was able to urinate 5 minutes later; his glans penis rapidly regained its normal color. Follow up after 3 months found no anomaly; notably, he had no urinary disorders or sexual dysfunction with respect to the International Index of Erectile Function.

### Patient 2

**Figure 2:**
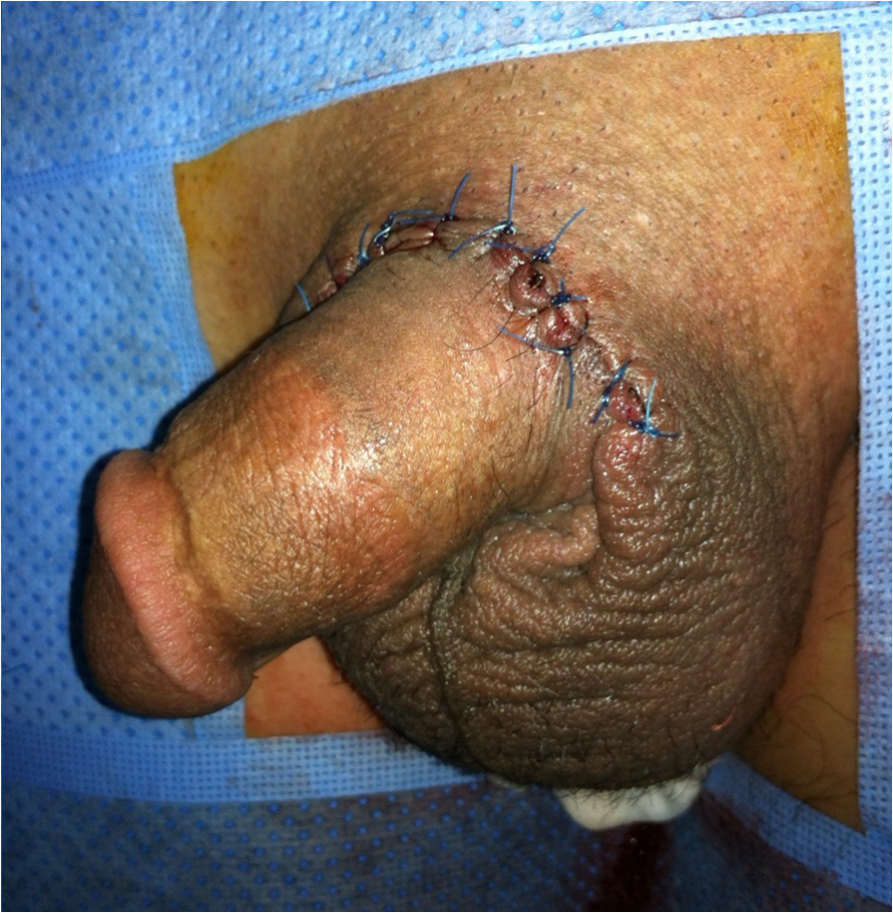
Appearance after the wound suture.

A 26-year-old Moroccan man without any relevant medical history was brought to our emergency unit by his family because of his scrotal pain and penile bleeding. A mental examination revealed that he was an aggressive patient with psychomotor agitation and heteroagressivity; he had visual and auditory hallucinations with hyperreligiosity. He explained that he mutilated his penis in order to protect himself from conspiracies around him by non-believers. A clinical examination found a wound on the dorsal surface of the root of his penis with complete transection of the dorsal vein of his penis and imperfect hemostasis. He was taken to the operating room for wound exploration. The exploration showed a complete transection of his dorsal vein which was ligated and a 1.5cm wound of the tunica albuginea that was repaired by an absorbable suture (Figure 2).

On the first follow up after 10 days, family members reported a remarkable improvement in his behavior. A clinical examination did not show any problems. A medical evaluation after 2 months showed that he had no voiding or erectile dysfunction problems.

Both patients were evaluated by a psychiatrist following the self-mutilation and the risk of suicide was considered low. Schizophrenia was diagnosed in both patients using the *Diagnostic and Statistical Manual of Mental Disorders*, fourth edition (DSM-IV), criteria. Their self-mutilation seemed justified by troubled thoughts arising from dark thoughts or hyperreligiosity. Other possible diagnoses such as mania and depression were ruled out.

The psychiatrist concluded that both patients had paranoid schizophrenia and they were given antipsychotic medication to which they responded well.

Nursing care in these cases was hectic especially in the first 2 days when the patients had not yet been stabilized under treatment. They required behavioral, pharmacological, and psychotherapeutic interventions to meet their highly complicated needs.

## Discussion

Self-mutilation is listed in the DSM-IV-text revision as a symptom of borderline personality disorder. However, patients with other diagnoses including those with depression, anxiety disorders, substance abuse, eating disorders, posttraumatic stress disorder, schizophrenia, and several personality disorders [3] can also self-harm.

The first genital mutilation case was published in 1846. A literature review since this period has shown an increase in incidence of this disorder [4] and a male predominance in genital self-mutilation [5], where castration and severe mental disorders predominate [6]. Greilsheimer and Groves [7] showed that in 87% of cases of self-mutilation a psychotic condition is seen in which 28.5% of patients are schizophrenic.

Eating behavior disorders (anorexia, bulimia) and genital self-mutilation have a common connotation of self-destruction. They have a self-purification function by modulating anxiety, sexual tension, anger or dissociation, providing an intense feeling of relief [8]. Self-mutilation is a way of expressing and dealing with deep distress and emotional pain. But the problem is that the relief that comes from self-mutilation does not last very long.

A common belief regarding self-mutilation is that it is an attention-seeking behavior; however, in most cases, this is inaccurate. Many self-harmers are very self-conscious of their wounds and scars and feel guilty about their behavior leading them to go to great lengths to conceal their behavior from others [9].

Large *et al.*[10] suggest that one of the primary causes for major self-mutilation is the individual’s first psychotic break. When individuals with schizophrenia engage in self-injury, the extent can be rather bizarre and potentially very harmful.

Persons with schizophrenia are known to attempt self-mutilation due to command hallucination, catatonic excitement or because of associated depression. Male genital self-mutilation in these cases has been reported by many authors [11].

Genital self-mutilation involves mutilation to the penis, the scrotum and the testicles. The type of injury varies from simple skin laceration (blade) to total amputation of the penis and testicles. Acute management of a patient who has engaged in serious self-mutilation should be tailored to the specific patient, but a series of general guidelines can be proposed. Repairing and reconstructing the penis remains a great challenge on anatomical, functional and aesthetical [12] aspects. First, repair or stabilization of the amputated limb or organ should be addressed. A decision to reimplant the organ must be made rapidly. Diagnosis of a severe penile trauma is made when two or more penile entities are injured: penile skin, corpora cavernosa, penile urethra or the glans penis. Patients should be transferred to a facility with microsurgical capabilities; however, if this is unavailable, macroscopic anastomosis of the urethra and corporeal bodies can be performed with good erectile results. Normal penile sensation returns in 0% to 10% of patients after macroscopic replantation, whereas sensation is present in more than 80% of microscopic reimplantations [13]. Adjuvant techniques after penile reimplant include the use of hyperbaric oxygen to promote healing or medical leeches on the penis after macroscopic replantation to increase venous outflow and decrease edema [13].

Concurrently, the patient must be evaluated at a psychiatric level which may complicate the decision-making process. Psychotic or agitated individuals commonly require sedation to allow adequate medical and surgical care [14].

Strangulation of the penis can cause ischemia causing necrosis that can occur during “perverse” handling. The first step is to remove the material responsible for the strangulation. Depending on the constricting device, significant resourcefulness maybe required from the physician. Apart from an obvious necrosis of the initial part of the penis, treatment should be as conservative as possible with an anti-inflammatory treatment to reduce edema [15].

If decompression is delayed and the patient is distressed and unable to void, a suprapubic bladder catheter should be placed. Outcomes are generally good with device removal alone, although the surgeon should be prepared to consider reconstructive techniques such as skin grafting if the strangulation injury caused skin necrosis [13].

## Conclusion

Cases of genital self-mutilation are urological and psychiatric emergencies, therefore it is important that surgical and psychiatric teams collaborate closely while managing cases of genital self-mutilation.

## Consent

As the patients were mentally incapable of making the decision, written informed consent was obtained from the legal guardians of the patients for publication of this case report and any accompanying images. Copies of the written consents are available for review by the Editor-in-Chief of this journal.

## Competing interests

The authors declare that they have no competing interests.

## Authors’ contributions

YK was the principal author and major contributor in writing the manuscript. DA, MA, AB, OR, RESW, SM, MFT, AK and IR analyzed and interpreted the patient data and reviewed the literature. MJE and MHF read and corrected the manuscript. All authors read and approved the final manuscript.
